# Histone acetylation and the role of histone deacetylases in normal cyclic endometrium

**DOI:** 10.1186/s12958-020-00637-5

**Published:** 2020-08-13

**Authors:** Palak Gujral, Vishakha Mahajan, Abbey C. Lissaman, Anna P. Ponnampalam

**Affiliations:** 1 The Liggins Institute, The University of Auckland, Auckland, New Zealand; 2 Department of Obstetrics and Gynaecology, Faculty of Medical and Health Sciences, The University of Auckland, Auckland, New Zealand; 3 Department of Physiology, Faculty of Medical and Health Sciences, The University of Auckland, Private Bag 92019, Auckland, 1142 New Zealand

**Keywords:** Histone acetylation, Histone deacetylation, Endometrium, Menstrual cycle

## Abstract

Histone acetylation is a critical epigenetic modification that changes chromatin architecture and regulates gene expression by opening or closing the chromatin structure. It plays an essential role in cell cycle progression and differentiation. The human endometrium goes through cycles of regeneration, proliferation, differentiation, and degradation each month; each phase requiring strict epigenetic regulation for the proper functioning of the endometrium. Aberrant histone acetylation and alterations in levels of two acetylation modulators - histone acetylases (HATs) and histone deacetylases (HDACs) - have been associated with endometrial pathologies such as endometrial cancer, implantation failures, and endometriosis. Thus, histone acetylation is likely to have an essential role in the regulation of endometrial remodelling throughout the menstrual cycle.

## Introduction

The human endometrium is a dynamic tissue. Its fundamental function is to provide an immunoprivileged site for embryo implantation and a nurturing environment for fetal development [1]. It mainly consists of luminal epithelium, glandular epithelium, and endometrial stromal cells; which undergo regeneration, proliferation, differentiation, and degradation under the influence of steroid hormones estrogen and progesterone [1–4]. There are several genes involved in cyclic morphological and functional changes in the endometrium. These are upregulated or downregulated depending on the stage of the menstrual cycle, suggesting tight regulation of gene expression in the endometrium [5].

Epigenetic modification is a critical regulator of gene expression and determinator of cellular properties. Histone acetylation is a primary epigenetic change that makes genes accessible or inaccessible to transcriptional factors and thus influences gene expression. Histone acetylation, in conjunction with other epigenetic modulators, has been associated with endometrial cyclic remodelling throughout the menstrual cycle; global histone acetylation levels have been seen to follow a cyclic pattern according to menstrual cycle stage in normal cyclic endometrium [6]. Histone acetylation is co-regulated by two sets of enzymes - histone acetyltransferases (HATs) and histone deacetylases (HDACs). Deregulation of HDACs and histone acetylation is often associated with endometrial pathologies such as cancer, endometriosis, and infertility [7–10]. However, there are very few studies explaining the role of histone acetylation and individual HDACs in endometrial stages and cell types. This review article summarizes the current literature on histone acetylation and the role of HDACs in normal cyclic endometrium and endometrial pathologies.

### Epigenetics

Epigenetic alteration refers to the chemical or a physical modification that affects gene accessibility, thus regulating how genes are read and expressed, without changing the DNA sequence [11]. These modifications are both heritable and reversible.

Some epigenetic modifications bring about life-changing alterations in an organism, while others are just part of normal cellular functions [12]. Epigenetic modifications work in conjunction with genetic regulation to determine cellular properties and functions.

Genes are epigenetically regulated by DNA methylation, chromatin remodelling, and non-coding RNAs [13]. DNA methylation silences by directly adding a methyl group to cytosine in the DNA sequence, chromatin remodelling regulates expression by tightening and loosening chromatin structure, and non-coding RNAs attach to complementary sequences resulting in silencing of that gene. These epigenetic modifications work in coordination with each other to regulate gene expression [14]. DNA methyltransferases (DNMTs) regulate the DNA methylation in cells. Chromatin remodelling is primarily carried out through covalent histone modifications by enzymes such as HATs, HDACs, methyltransferases, and kinases [15]. Non-coding RNAs - including siRNAs, miRNAs, piRNAs, and long non-coding RNAs - epigenetically modify the genes by inducing transcriptional gene silencing [13].

Emerging research in epigenetics suggests that a range of environmental factors, lifestyle, early life stress, and trauma can influence the establishment and maintenance of epigenetic marks through generations (often known as epigenetic memory) [11, 16, 17]. Epigenetic reprogramming of induced pluripotent stem cells to develop novel treatments for diseases is an emerging area of research [18]. Epigenetic regulation is required for normal cellular functions; however, some epigenetic modifications can also lead to conditions such as cancer, developmental disorders, genomic imprinting disorders, X-chromosome inactivation and endometriosis [19–21].

### Histone modifications

**Fig. 1:**
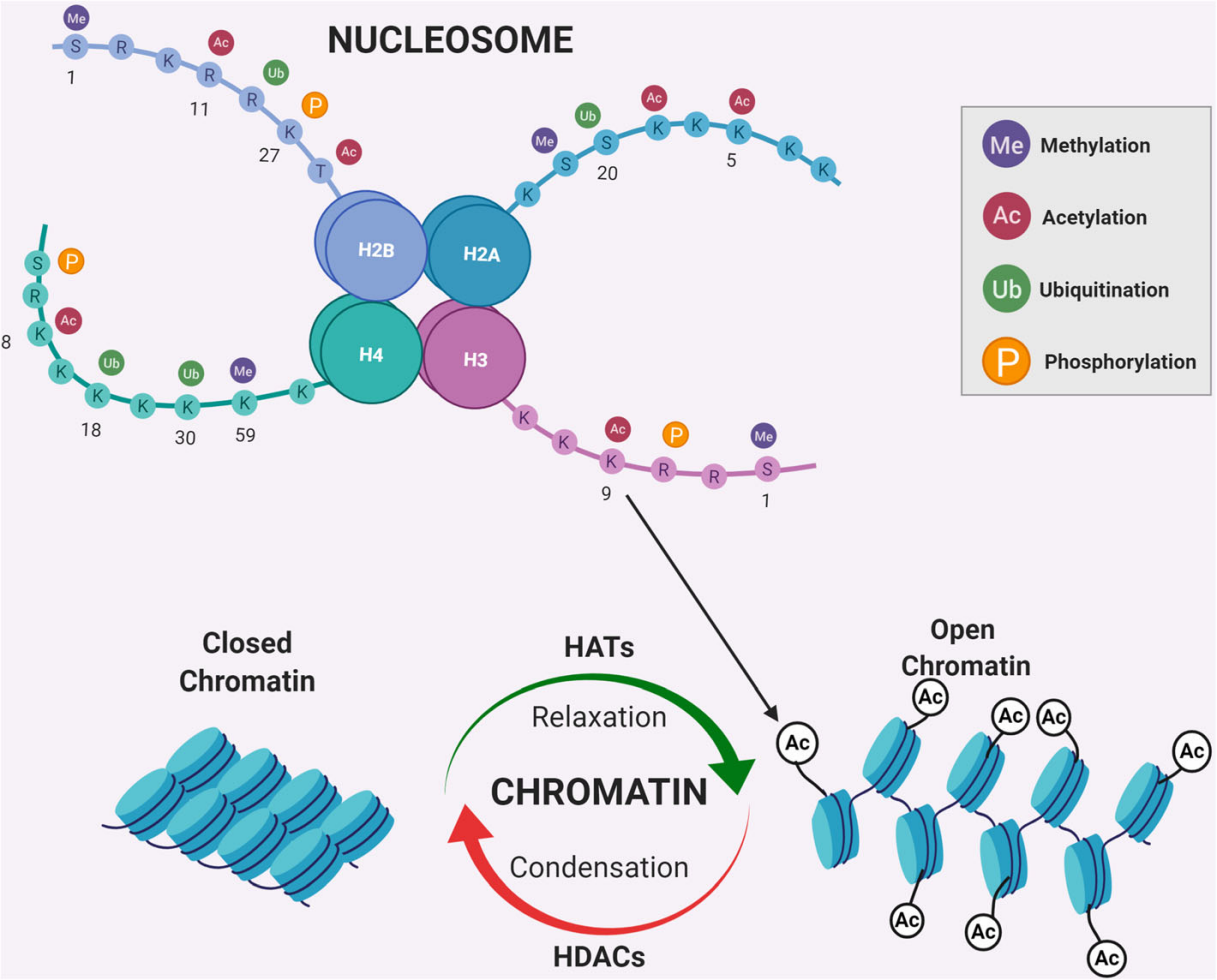
Chromatin remodelling*.* Fundamental structure of chromatin called nucleosome consists of two sets of four histone proteins H2A, H2B, H3 and H4. Protruding histone tails undergo post translational modifications such as methylation, acetylation, ubiquitination and phosphorylation. The numbers indicate the positions of targeted lysine groups. Histone acetylation alters the conformation of chromatin structure in nucleus by relaxing the chromatin and allowing transcriptional activation. It is regulated by two sets of enzymes HATs and HDACs which add or remove acetyl group respectively from both histone and non-histone proteins, hence regulating gene transcription. (Created with BioRender.com)

Approximately 2 m of DNA is packaged inside a nucleus with the help of proteins called histones, forming a DNA-protein complex called chromatin. The fundamental unit of chromatin is called the nucleosome, which consists of two sets of the four core histones – H2A, H2B, H3 and H4 [22] (Fig. 1). Histone H1 links the nucleosomes together to form a chromosome [23]. This structure goes through many enzymatic modifications in-vivo, which gives rise to several genetic variabilities [24]. Histones have a protruding tail, which is subject to post-translational modifications (PTMs), such as acetylation, phosphorylation, lysine methylation, arginine methylation, deamination, ADP ribosylation, proline isomerization, β-N-acetylglucosamine, ubiquitylation, and sumoylation [25, 26]. Histone modification regulates chromatin structure; facilitates remodelling; and affects DNA processes such as replication, repair, and recombination [25]. Most of these histone modifications are reversible and provide scope for developing novel therapeutic methods.

### Histone acetylation

First identified in 1964 [27], histone acetylation involves the addition of an acetyl group to lysine residues in the protruding histone tails [25, 28]. It is usually associated with transcriptional activation, and is modulated by two opposing groups of enzymes; histone acetyl transferases (HATs), which are responsible for adding acetyl groups; and histone deacetylases (HDACs), which remove them [25] (Fig. 1).

#### Histone acetyltransferases

HATs add an acetyl group to the ε-amino group of lysine using acetyl CoA as a cofactor, which neutralizes the positive charge on lysine, weakens the histone-DNA interaction, and makes genes accessible [25].

HATs are a diverse set of proteins. So far, about 30 HATs have been identified in humans. HATs are primarily classified into two classes based on their subcellular localization: Type A and Type B. Type A HATs are localized in the nucleus, while Type B is found in the cytoplasm. The Type A HATs function in transcription-related histone acetylation in chromatin, while Type B HATs acetylate newly synthesized histones and influence the structure of the nucleosome [29, 30].

Type A HATs are further grouped into five families based on their catalytic domain [31, 32]. The Gcn5-related N-acetyltransferase (GNAT) family includes P300/CBP-associated factor (PCAF), Gnc5, and ELP3; the MYST family includes Tip60, monocytic leukemic zinc finger (MOZ), MOZ-related factor (MORF), human acetylase binding to ORC1 (HBO1), and human-males-absent-on-the-first (HMOF) [32, 33]; the CBP/p300 family includes CBP and p300 [32, 34], and the transcription factor related family includes TAF1 and TIFIIIC90. Besides these, HATs also include many steroid receptor co-activators [32, 35, 36].

Deregulation of HATs is associated with cancer formation [37]. CBP/p300 is essential for the transition from G1 to S stage in the cell cycle and can function as either a tumor suppressor or an oncogene depending on its localization [38, 39]. Selective inhibition of p300 inhibits the cell cycle and induces apoptosis. It inhibits the response to DNA damage in melanoma cells [40]. MYST family HATs have various roles in stem cell function and development [33]. Because HATs are reversible regulators and are involved in cell cycle progression, they are being studied as targets for tumor growth management [41].

#### Histone deacetylases

HDACs are post-transcriptional modulators that remove acetyl groups from lysine residues of both histone and nonhistone proteins [42]. In humans, 18 HDACs have been identified and grouped into four classes based on their sequence homology to yeast [42, 43]: Class I (HDAC 1, 2, 3, and 8); class II (HDAC 4, 5, 6, 7, 9 and 10); class III (SIRT 1, 2, 3, 4, 5, 6 and 7) and class IV (HDAC 11). Class, I, II, and IV HDACs are Zn^2+^ dependent enzymes and have similar functional mechanisms whereas class III HDACs are NAD+ dependent. HDACs have relatively low substrate specificity, and one HDAC can act on multiple substrates or multiple HDACs can act on the same substrate [42]. Additionally, they work in the form of complexes with other HDACs and enzymes. These complications make it difficult to interpret the individual functions of HDACs [25]. There is strong evidence of cross-talk between HDACs and other epigenetic factors in the regulation of cancer tumorigenesis [44].

HDACs have proved to be dynamic enzymes that can modify a variety of proteins. The involvement of HDACs in regulating fundamental cellular functions such as proliferation, cell cycle, regeneration, apoptosis, and differentiation makes them an important target of study for disorders [43].

Class I HDACs are expressed in all tissues. HDAC 1 and 2 have been involved in cellular functions such as proliferation and apoptosis, while HDAC 3 is involved in the DNA damage response [43, 45]. HDACs 4, 5, 7, and 9 are associated with cell differentiation and development [45]. Sirtuins are mostly involved in cellular metabolism and DNA repair, while HDAC 11 is involved in regulating the expression of interleukins [46, 47]. Deregulation of HDACs is usually associated with cancer and tumorigenesis. Many HDAC inhibitors are now FDA approved anticancer drugs [48].

### The endometrium

#### The menstrual cycle

Each month the endometrium goes through controlled cyclic changes, which are divided into three major phases: proliferative phase, secretory phase, and menstruation. This is called the menstrual cycle, and it occurs approximately 400 times in a lifetime [49].

**Fig. 2:**
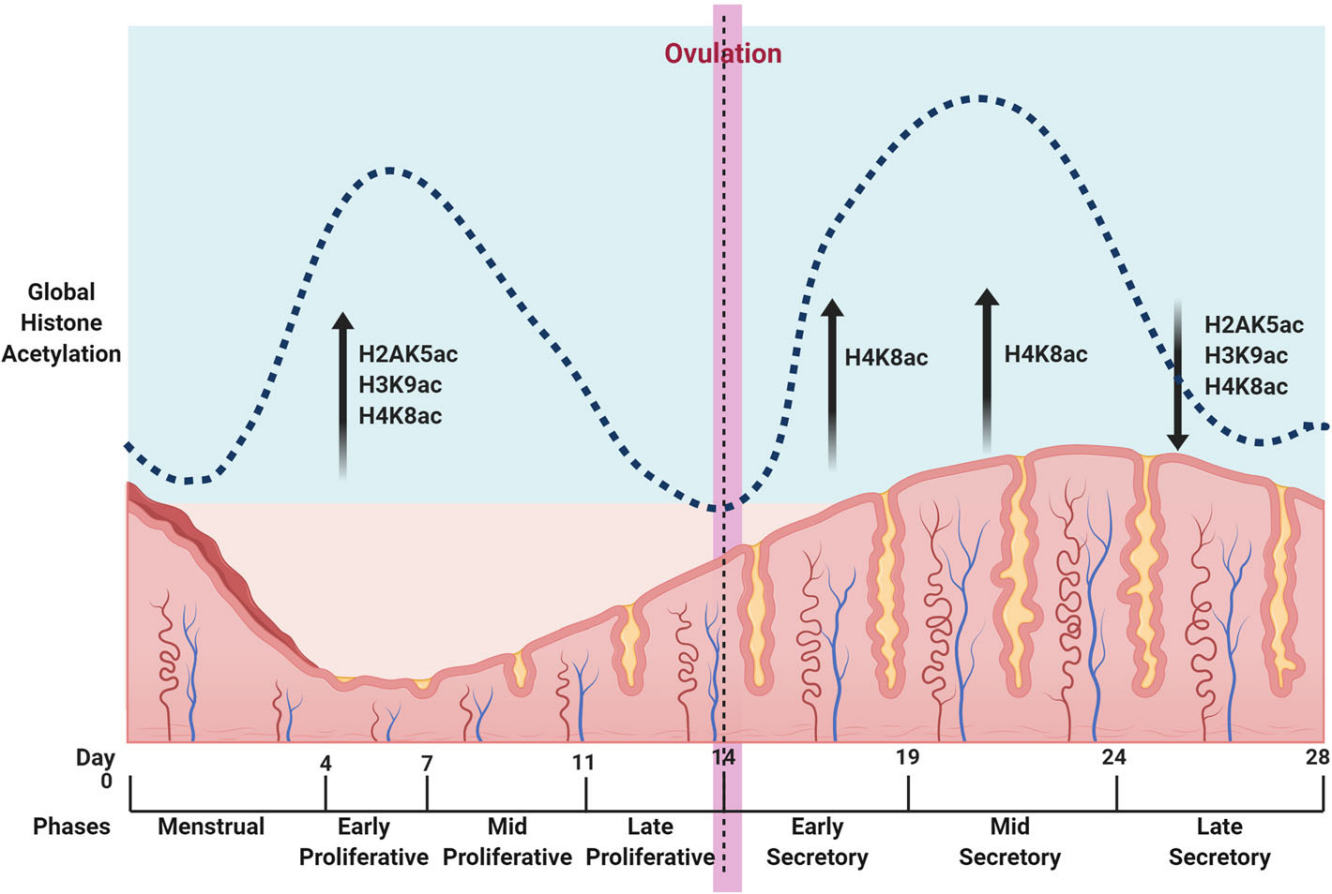
Global histone acetylation changes throughout the menstrual cycle*.* Global histone acetylation changes have been observed in human endometrium during different stages of menstrual cycle. Acetylation levels of H2AK5, H3K9, and H4K8 are elevated during early proliferative phase, the acetylation levels decline subsequently until ovulation then a significant increase in acetylation levels of H4K8 is observed in early secretory phase which reaches its peak during mid secretory phase. A significant decline is observed in global histone acetylation levels in H2AK5, H3K9, and H4K8 during late secretory phase [6]. (Created with BioRender.com)

The proliferative phase starts around day 5 of the menstrual cycle and ends around day 14 [50]. The proliferative phase of the endometrium runs parallel to the ovarian follicular cycle, and it is regulated by estrogen secreted from the developing ovarian follicle. The rising levels of estrogen help restore the funtionalis layer of the endometrium [51], leading to the thickening of the endometrium, growth of endometrial glands, and emergence of spiral arteries [52]. The global acetylation levels of H2AK5, H3K9, and H4K8 in human cyclic endometrium have been seen to be elevated in the early proliferative phase [6]. This may be associated with the requirement of transcriptional activation of regenerative and proliferative pathways during the early proliferative phase. The acetylation levels decline in later stages until ovulation, suggesting a switch from proliferative to secretory functions [6] (Fig. 2).

Post ovulation, around day 14 of the menstrual cycle, there is an increase in progesterone levels produced by the corpus luteum, which gives rise to secretory changes in the endometrium [53]. The first half of the secretory phase involves glandular changes; stromal changes are more evident during the second half [53]. Spiral arteries and glands become tortuous during this phase [2]. The endometrial cells go through morphological and functional changes to differentiate and prepare for implantation, a process called decidualization. The human endometrium is decidualized in each menstrual cycle [54]. A significant increase in acetylation levels of H4K8 has been seen post ovulation, and a similar trend has also been observed in H3K9 and H4K14/18 (Fig. 2). This correlates with the requirement for secretory modifications like cellular differentiation, angiogenesis, and decidualization [6].

In the absence of a fertilized embryo, the fall in progesterone levels triggers the breakdown of the decidualized endometrial lining. This process is called menstruation [2], and correlates with the decline in histone acetylation levels seen in the late secretory phase [6] (Fig. 2).

Interestingly, other estrogen and progesterone responsive tissues such as breast, fallopian tubes, and vagina do not shed in response to the decline in hormonal levels [2]. After menstruation, the endometrial lining starts to regenerate and repair to prepare for the next cycle.

These perfectly timed events of regeneration, proliferation, differentiation, and degradation require strict epigenetic and genetic regulation of endometrial remodelling. Many endometrial pathologies are associated with the deregulation of epigenetic control. Understanding the epigenetic regulation of the endometrial lining is also essential in order to improve artificial reproductive technologies by identifying the receptive endometrium and modulating the endometrial receptivity state [55].

#### Regeneration

After each menstrual cycle, the endometrial functionalis layer regenerates from the stratum basalis. It grows from an initial thickness of 0.5–1.0 mm to up to 7.0–8.0 mm in the secretory phase [53]. It not only regenerates during the menstrual cycle, but also following parturition; almost complete resection; and in postmenopausal women taking hormone replacement therapy [56, 57]. Endometrial re-epithelialization in humans occurs rapidly and is scar-free [58]. For several years, epigenetic regulation has been linked to regeneration processes [59]. HDAC inhibitors or histone acetylating agents are capable of inducing scar-free wound healing by stimulating cytokines or growth factors, which are crucial for rapid re-epithelialization [60].

The remarkable regenerative capacity of the endometrium is due to the presence of adult progenitor stem cells [61]. These cells are found throughout the body and play a crucial role in the regeneration and repair of damaged tissues and the maintenance of organs. Epithelial progenitor cells and mesenchymal stem/stromal cells play an essential role in the regeneration and repair of endometrial epithelium and stroma [56]. A small population of mesenchymal stem cells (MSC) - a subpopulation of adult progenitor stem cells - can be found in the endometrium and are called endometrial MSCs (eMSC) [62, 63]. They are highly regenerative stem cells that have similar properties to bone marrow MSCs [56]. Menstrual blood also contains clonogenic, multipotent MSCs, which have a broad differentiation capacity [64].

Over the past decade, several studies have shown that epigenetics are a crucial regulator of stem cell functioning [65]. Histone modification changes chromatin architecture and regulates gene expression. Since histone acetylation is an essential post-transcription modulator, it is a basic requirement in stem cell functioning and works in coordination with DNA methylation activity. The fate determination of MSCs is controlled by a complex network of transcription factors and histone-modifying enzymes [66]. HDAC 1 silencing improves the efficiency of human umbilical cord MSCs in mouse models with traumatic brain injury [67]; while HDAC 6 deficiency, causing acetylation of p53 K120, can induce apoptosis in MSCs [68]. Global H3K9Ac level decreases, and H3K9Me2 increases during osteogenic differentiation of MSCs [69]. These studies indicate the involvement of histone acetylation in the regulation of MSC function.

The endometrium has become a popular source of stem cells for reprogramming into induced pluripotent stem cells (iPSCs) [56]. The MSCs derived from the endometrium and umbilical cord are being used as iPSCs in regenerative medicine, exploiting the epigenetic regulation of these cells [70, 71]. HDAC inhibitors such as valproic acid (VPA), trichostatin A (TSA), suberoylanilide hydroxamic acid (SAHA), and butyrate have been known to enhance reprogramming in iPSCs [72].

Aberrant epigenetic regulation in eMSCs has been associated with endometriosis pathogenesis [73]. eMSCs are being targeted for the development of novel therapies for the treatment of endometriosis via epigenetic reprogramming [74].

Upregulation of the global acetylation levels of H2AK5, H3K9, and H4K8 during the early proliferative phase in the adult endometrium correlates with transcription activation of several regenerative pathways [6]. Although changes in histone acetylation levels and HDACs in normal to diseased conditions have been discussed, and known evidence of epigenetic involvement in endometrial regeneration is available, the target genes and level of HDAC expression throughout the menstrual cycle have not yet been identified.

Histone acetylation and DNA-methylation work in a coordinated manner, decide stem cell fate and influence cancer pathogenesis [75].

#### Proliferation

After endometrium regeneration, estrogen levels from the developing follicle stimulate the proliferation of stromal and epithelial cells during the proliferative phase of the menstrual cycle [56, 76]. Estrogen and progesterone surface receptors increase in number, and the rapid formation of new blood vessels, called angiogenesis, occurs to support the nutritional requirements of the growing tissue [77].

Cancer studies provide us with great insight into the epigenetic regulation of cell proliferation. HDACs can act as both suppressors and inducers of cell proliferation, depending on the gene they regulate. Inhibition of HDAC 1 reduces cell proliferation, and inhibition of HDAC 3 is associated with decreased migration of ovarian cancer cells [78].

Class I HDACs [1–3, 8] are critical for modulating cell survival and proliferation, and HDACs 1, 2, and 3 play an important role in steroid hormone-dependent gene expression in the human endometrium [9, 10]. A previous study on human cyclic endometrial tissues has demonstrated that the global acetylation levels of H2AK5, H3K9, and H4K8 are elevated in the early proliferative phase, and are associated with the transcriptional activation of proliferative and regenerative pathways [6]. HDAC expression is upregulated in most endometrial cancers compared to normal cyclic endometrium [79]. A study on endometrial stromal sarcoma (ESS) showed that expression of HDACs 1 and 2 is higher in ESS compared to non-neoplastic stem cells, with HDAC 2 expression being slightly higher than HDAC 1. Studies also show that inhibition of HDAC 2 can lead to cell differentiation and inhibition of proliferation [80, 81]. HDAC inhibitors can induce expression of p21 and p27 (endogenous cyclin-dependent kinase inhibitors), which cause cell cycle arrest and inhibit proliferation [82]. Several studies have used HDAC inhibitors as anti-cancer agents to normalize cell proliferation in endometrial carcinomas [10, 79, 83–85]. SAHA, m-carboxycinnamic acid Bishydroxamide (CBHA), Scriptaid, Oxamflatin, VPA, Sodium Butyrate, M344, Apicidine, Psammaplin A (PsA) and MS-275 are HDAC inhibitors that have been successfully studied in endometrial cancers and are found to induce cell cycle arrest and regulate proliferation [79].

Studies using HDAC inhibitors VPA and SAHA have also shown the association between HDACs and angiogenesis in vitro*.* The study showed that treatment with HDAC inhibitors VPA and SAHA, in combination with vascular endothelial growth factor (VEGF), increased endothelial cell sprouting [86], suggesting an association between histone acetylation and angiogenesis. Because angiogenesis is crucial for endometrial repair and re-epithelialization [87], the role of histone acetylation in angiogenesis is critical for menstrual cycle regulation.

#### Decidualization and implantation

The rise in progesterone level post ovulation gives rise to morphological and functional changes in the human endometrium called decidualization. These changes include differentiation of fibroblastoid endometrial stromal cells (ESC) into decidual cells, the presence of decidual white blood cells, and vascular modifications in maternal arteries [54, 88]. Decidualization initiates a cascade of events that allow the embryo to attach to the endometrium and coordinates the trophoblast invasion [89]. Invasion of trophoblast into endometrial decidua requires tissue remodelling enzymes [90]. Matrix metalloproteinases (MMPs) are a group of enzymes required for tissue remodelling and degrading extracellular matrix (ECM) [91, 92]. During implantation and trophoblast invasion, MMP-2, and MMP-3 are expressed and secreted by human endometrium. In contrast, the decidual stromal cells express tissue inhibitors of MMPs (TIMPs) that limit the degradation of ECM by MMPs, thus hindering trophoblast invasion. A balance between MMPs and TIMPs in the endometrium is required for successful implantation of the blastocyst [91, 93, 94].

Decidualization, being a complicated process, requires dramatic gene expression changes in ESCs [88]. These changes are associated with histone modifications, and several studies from the past two decades have shown a correlation between decidualization and histone modifications. In the previous study by *Munro* et al.*,* on human endometrial tissue biopsies, a statistically significant increase in H4K8 expression was seen, and H3K9 and H4K14/18 followed a similar trend [6]. These findings correlate well with another study on ESCs cultured with estrogen and progesterone, which showed a significant increase in the acetylation of H4K8 and H3K9/14 [95]. In a genome-wide analysis of histone modifications in human ESCs, H3K27ac and H3K4me3 levels were high during decidualization. Genes with increased H3K27ac and H3K4me3 levels were found to be involved in insulin signaling pathways, crucial for the decidualization process [88].

Trichostatin A (TSA) is an inhibitor of HDAC and is involved in the inhibition of HDACs class I, II, and IV; TSA however, does not inhibit class III HDACs (Sirtuins) [96]. A comparative study between cultured ESCs and glandular cells isolated from human endometrium showed that the addition of TSA enhances the upregulation of decidualization markers such as insulin-like growth factor binding protein-1 (*IGFBP-1*) and *prolactin*, as regulated by 17β-estradiol (E2) plus progesterone (P4) [95]. This study showed the involvement of histone acetylation and the connection of HDACs to the decidualization of the endometrium, suggesting that TSA acts as an enhancer of the decidualization process. In contrast, a decade later, another study showed that treatment of human ESCs with TSA had an inhibitory effect on trophoblast invasion. The ESCs treated with TSA showed increased *TIMP-1* and *TIMP-3* expression, while expression of *MMPs* was decreased. This study associated histone acetylation with the disruption of trophoblast invasion and suggested HDACs are critical for implantation [91]. A recently published study on mice demonstrated that loss of HDAC 3 in the uterus of mice leads to implantation failures. Interestingly, HDAC 3 is usually the least frequently expressed HDAC in the endometrial tissue [7, 10]. In the study, HDAC 3 was shown to be involved in transcription activation of *COL1A1* and *COL1A2* (collagenase) genes in humans and *col1a1* and *col1a2* genes in mice [7]. Collagenases are involved in endometrial remodelling and trophoblast invasion during implantation [91]. The cumulative findings of these studies suggest that HDAC 3 could be crucial for the implantation process, but in a limited capacity. A tightly controlled relationship between HATs and HDACs is essential for proper decidualization and successful implantation.

### Histone acetylation and endometrial pathologies

The highly timed molecular events discussed above, if not appropriately regulated, lead to a spectrum of endometrial pathologies such as endometriosis, endometrial cancers, abnormal uterine bleeding, and infertility.

**Fig. 3:**
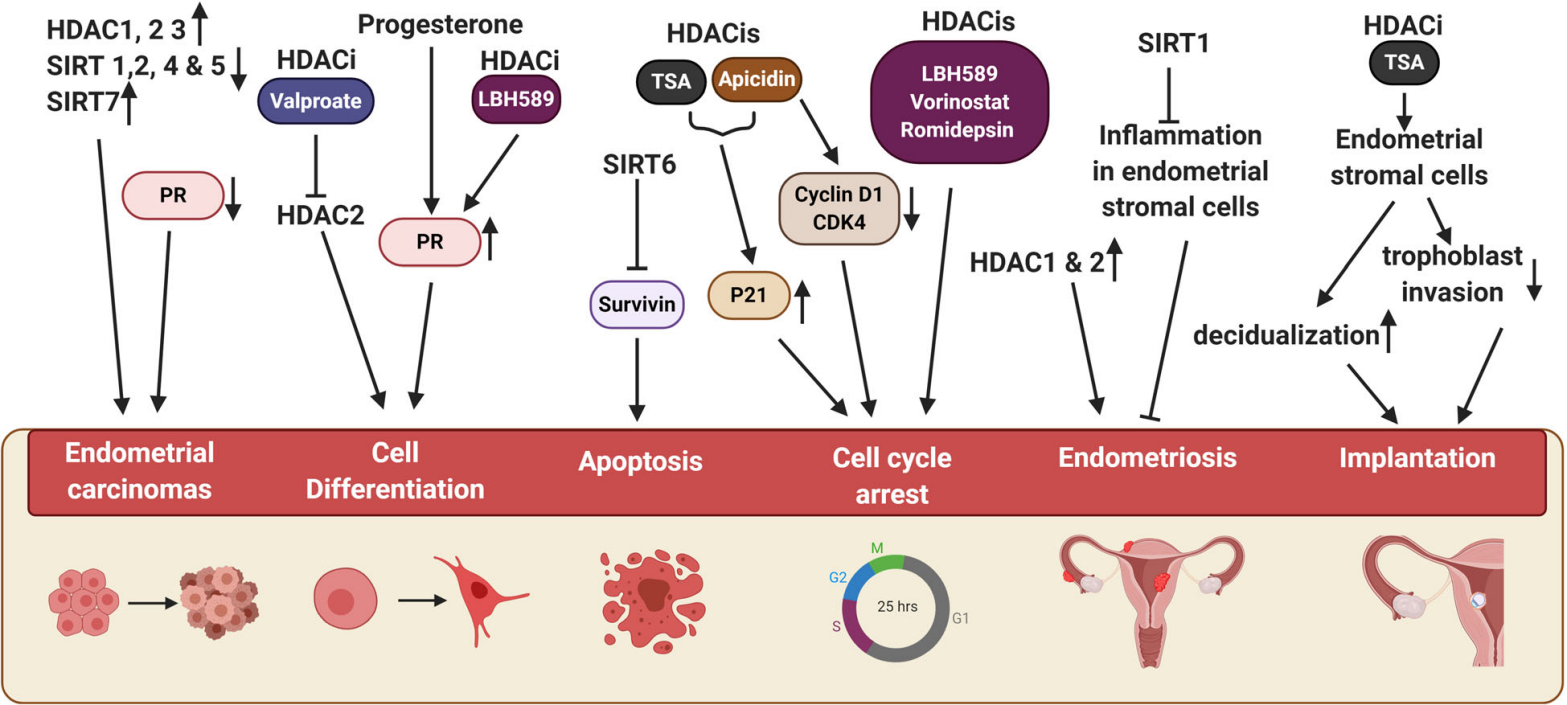
Potential effects of HDACs and HDAC inhibitors (HDACis) observed in endometrial pathologies to date*.* HDACs and HDAC inhibition with HDACis influence the endometrium either directly or indirectly. Several HDACs are differentially expressed in endometrial carcinomas [85, 103]. Inhibition of HDAC2 by valproate induces endometrial cell differentiation [80]. Studies in endometrial carcinoma cells in vitro imply that progesterone through PR induces cell differentiation, while HDACi LBH589 increases *PR* expression in endometrial cells [101, 102].; SIRT6 induces apoptosis by acting on survivin [103]; TSA and apicidin treatment increase P21 expression, while apicidin alone induces cell cycle arrest by reducing cyclin D1 and CDK4 expression [81, 98]. Cell cycle arrest can also be caused by Vorinostat, Romidepsin, and LBH589 (99–101). HDAC1 and 2 expression levels are upregulated in endometriosis in vitro [8]. and inhibition of SIRT1 can trigger inflammatory response in endometriotic stromal cells [105]. Inhibition of HDACs with TSA induce decidualisation in stromal cells while controlling trophoblast invasion, in vitro [91, 95]. (Created with BioRender.com)

Studies have found the involvement of histone acetylation, in conjunction with other epigenetic modulators, in several human malignancies. High expression of class I HDACs in some tumors has been linked to unfavorable prognosis, with the exception of breast cancer in which it has a favorable prognosis. On the other hand, the upregulation of class II HDACs is associated with a favorable prognosis in human tumors [97]. One of the earliest studies on endometrial stromal sarcoma (ESS) showed that HDAC 2 expression is upregulated in ESS compared to the non-neoplastic endometrial stroma [80]. They also showed that HDAC 1 expression is generally lower than HDAC 2, and inhibition of HDAC 2 using valproate affects cell differentiation by increasing the expression of cell cycle regulators [80]. Later, a study on endometrial adenocarcinomas showed a decrease or complete loss of epithelial HDAC 1 protein expression compared to normal endometrium [10]. Alternatively, a study on endometrial carcinomas (EC) showed that HDAC 1, 2 and 3 expression levels are increased in ECs compared to healthy endometrial tissues [85]. Although this study did not account for the cycle stages of the endometrium in analysis, it still showed similarity with earlier studies, in that HDAC 2 is the most expressed in the endometrium, while HDAC 3 is the least expressed [10, 85]. An immunohistochemical study on endometrial carcinoma showed that expression of HDAC 1, HDAC 2, and Ki-67 (a cellular marker of proliferation) are higher in endometrial carcinomas than in normal endometrium. The treatment of endometrial carcinoma cell lines with HDAC inhibitors TSA and apicidin reduces cell proliferation and increases p21 expression. Apicidin also reduces cyclin D1 and CDK4 expression [81, 98]. HDAC inhibitors Vorinostat, Romidepsin (FK228), and LBH589 help induce cell cycle arrest in endometrial carcinomas [98–100]. Progesterone induces differentiation in endometrial cells and inhibits proliferation through the action of progesterone receptors (PR). Therefore, the loss of PR also leads to endometrial carcinomas. Treatment of ECC-1 and Ishikawa H cells with the HDAC inhibitor LBH589 has been shown to increase *PR* expression, which in turn regulates cell differentiation and induces cell cycle arrest [101, 102]. While most HDACs are upregulated in ECs, recent studies have shown that SIRT 6 induces apoptosis and inhibits proliferation in endometrial cancer cells by repressing survivin [103]. Expression levels of *SIRT 1*, *SIRT 2*, *SIRT 4*, and *SIRT 5* are seen to be downregulated, while *SIRT 7* is significantly upregulated in ECs compared to non-neoplastic cells. Interestingly, this study showed no significant difference in *SIRT 6* and *SIRT 3* levels between ECs and non-neoplastic cells [104] (Fig. 3).

Histone acetylation and aberrant levels of HDACs are also associated with endometriosis. HDAC 1 expression is seen to be significantly elevated during endometriosis compared to normal endometrium, and the level also correlates with low acetylation levels of H3 and H4 [9, 106]. It was seen again in a later study that HDAC 1 and 2 expression levels were high in endometriotic stromal cells compared to healthy endometrial stromal cells. Differential expression of HDAC 1 and 2 was observed based on lesion type and localization in endometrioid cells [8]. A comparative study between ectopic and eutopic endometrial samples taken from women who have endometriosis showed that *HDAC 1* gene expression was high in ectopic tissues while *HDAC 2* expression levels were high in eutopic tissue samples. Expression of HATs *P300* and *CREBBP* did not change significantly when endometriosis tissue samples were compared to standard samples, while *PCAF* expression was high in the ectopic tissue [107]. It should be noted that this study used total tissue samples with minimal sample size, and the total sample contained varied cell populations from various endometrial cycle stages. The anti-inflammatory effects of SIRT1 have also been investigated in endometriosis. This study compared the action of SIRT1 and its activator resveratrol in endometriotic stromal cells and healthy endometrial stromal cells. They found that the activation of SIRT1 suppresses inflammatory responses in endometriotic stromal cells, while inhibition of SIRT1 can trigger inflammatory responses. Suggesting its crucial role in maintaining healthy endometrial stromal cells [105] (Fig. 3).

HDACs also play a crucial role in implantation and decidualization. Loss of *HDAC 3* is linked to decidualization defects and implantation failure in mice [7]. HDAC inhibitor TSA in endometrial stromal cells negatively regulates trophoblast invasion and facilitates decidualization, influencing embryo implantation [91, 95] (Fig. 3).

Since most of the studies on endometriosis and other endometrial pathologies compare healthy and diseased tissue samples, it is hard to tell if the epigenetic aberrations are the cause or effect of various endometrial pathologies.

## Conclusions

Histone acetylation is a fundamental regulator of chromatin structure and gene expression. Many studies on endometrial tissues and cell lines have shown the involvement of aberrant levels of histone acetylation and HDACs in endometrial pathologies, especially endometrial carcinomas and endometriosis. The majority of HDACs studied seem to be elevated in endometrial carcinomas compared to the normal endometrium [8, 85]. High levels of HDACs are also associated with endometriosis in many women. Furthermore, evidence suggests that histone acetylation and HDACs are involved in various endometrial pathologies [7–9, 79, 83]. However, most of these studies have not given any regard to the cyclic nature of the endometrium. HDACs are being studied as potential therapeutic targets for endometrial carcinomas and endometriosis; their effect reversed with HDAC inhibitors [79, 83]. Global histone acetylation levels of H2AK5, H3K9, and H4K8 are found to be elevated in the early proliferative phase of the endometrium, then decline until ovulation. The acetylation levels in H4K8 rise significantly post ovulation, and a similar trend is observed in H3K9 as well as H4K14 and 18 (Fig. 2). This study suggests that histone acetylation trends seem to follow a cyclic pattern in coordination with menstrual cycle events that require transcriptional activation and silencing [6]. HDACs function in conjunction with other epigenetic modulators, regulating molecular modifications in the endometrium [107]. Studying the general levels of HDACs and histone acetylation in normal cyclic endometrium will give us insight into individual functions and targets of individual HDACs. Because histone acetylation is a reversible epigenetic mark, studying its regulation will eventually help us to develop targeted therapies to improve the menstrual health of women.

## Data Availability

Not applicable.

## Abbreviations

DNMT: DNA methyltransferase
EC: Endometrial carcinomas
ECM: Extracellular matrix
eMSC: Endometrial mesenchymal stem cells
ESC: Endometrial stromal cell
ESS: Endometrial stromal sarcoma
FDA: U.S. Food and Drug Administration
GNAT: Gcn5-related N-acetyltransferase
HAT: Histone acetyltransferase
HBO1: Human acetylase binding to ORC1
HDAC: Histone deacetylase
HDACi: Histone deacetylase inhibitor
HMOF: Human-males-absent-on-the-first
iPSC: Indiced pluripotent stem cells
MMP: Matrix metalloproteinases
MORF: MOZ related factor
MOZ: Monocytic leukemic zinc finger
MSC: Mesenchymal stem cells
PCAF: P300/CBP-associated factor
PR: Progesterone receptor
PTM: Post-translational modification
SAHA: Suberoylanilide hydroxamic acid
TIMPS: Tissue inhibitors of MMPs
TSA: Trichostatin A
VEGF: Vascular endothelial growth factor
VPA: Valporic acid
